# Lipoprotein(a) serum concentrations in children in relation to body mass index, age and sex

**DOI:** 10.1038/s41390-024-03108-4

**Published:** 2024-02-28

**Authors:** Paulina E. Stürzebecher, Konstantin L. Uttinger, Mandy Vogel, Maike Schlingmann, Uta Ceglarek, Berend Isermann, Wieland Kiess, Antje Körner, Ulrich Laufs

**Affiliations:** 1 Klinik und Poliklinik für Kardiologie, Universitätsklinikum Leipzig, Leipzig, 04103 Germany; 2 Department of Visceral, Transplant, Thoracic and Vascular Surgery at Leipzig University Hospital, Leipzig, Germany; 3 LIFE Leipzig Research Center for Civilization Diseases, University of Leipzig, 04103 Leipzig, Germany; 4 Hospital for Children and Adolescents and Center for Pediatric Research (CPL), University of Leipzig, Liebigstrasse 20a, 04103 Leipzig, Germany; 5 Institute of Laboratory Medicine, Clinical Chemistry and Molecular Diagnostic, University Hospital Leipzig, 04103 Leipzig, Germany

## Abstract

**Background:**

Lipoprotein(a) (Lp(a)) is an inherited risk factor for atherosclerotic cardiovascular disease (ASCVD). Limited data exist on Lp(a) values in children. We aimed to evaluate whether Lp(a) concentrations in youth are influenced by BMI.

**Methods:**

756 blood samples of 248 children with obesity and 264 matched healthy children aged 5 and 18 years, enrolled in the population-based LIFE Child (German civilization diseases cohort) study, were analyzed. Repeat measurements were available in 154 children (1–4 follow ups, ~1 year apart).

**Results:**

The median Lp(a) concentration in the total cohort (*n* = 512) at first visit was 9.7 mg/dL (IQR 4.0–28.3). Lp(a) concentrations between 30–50 mg/dL were observed in 11.5%, while 12.5% exhibited Lp(a) ≧50 mg/dL. There was no association of Lp(a) with body mass index (BMI) (ß = 0.004, *P* = 0.49). Lp(a) levels did not correlate with age or sex, while Lp(a) was associated positively with low-density lipoprotein cholesterol (ß = 0.05, *P* < 0.0001). The Lp(a) risk category remained stable in 94% of all children in repeated measurements.

**Conclusions:**

The data showed no association of Lp(a) levels in children with BMI, age or sex. Measurement of Lp(a) in youth may be useful to identify children at increased lifetime risk for ASCVD.

**Impact:**

- In youth, Lp(a) levels are not affected by age, sex and BMI.
- Lp(a) risk categories remain stable over time in repeated measurements in children.
- Measurement of Lp(a) in children may be useful as an additional factor to identify children at increased lifetime risk for ASCVD and for reverse family screening.

## Introduction

High lipoprotein(a) (Lp(a)) serum concentrations are an independent risk marker of atherosclerotic cardiovascular disease (ASCVD) and aortic valve stenosis in adults.^1,2^ Lp(a) exerts pro-inflammatory and pro-atherogenic effects, increasing the risk for early onset of ASCVD and aortic valve stenosis.^3,4^ Up to 90% of the variation in Lp(a) plasma levels is genetically determined, and Lp(a) plasma levels are minimally impacted by lifestyle or oral lipid-lowering therapies.^3^ In adults, a single measurement is recommended to determine an individual’s Lp(a) associated risk: <30 mg/dL for no risk, 30- <50 mg/dL for the interim gray zone, and ≥50 mg/dL for clear Lp(a) associated risk.^5,6^ Lp(a) has emerged as an excellent parameter for risk stratification and for family screening in adults.^5^ Thus, the current guidelines of the European Society of Cardiology (ESC) and the European Atherosclerosis Society (EAS) recommend measuring Lp(a) in every individual once in a lifetime.^6,7^ In contrast to adults, information on Lp(a) serum concentration in children is limited, and it is unknown whether Lp(a) screening in children would likewise identify individuals at increased risk of ASCVD.^8–12^ Early detection and treatment of dyslipidemias are crucial for the primary prevention of lifelong exposure of vessel walls to pathogenic lipoproteins, preventing the development of ASCVD that likely starts in childhood.^13,14^ There is accumulating evidence that elevated Lp(a) levels are present from an early age.^10,11,15^

One subgroup of children which is especially vulnerable to cardiovascular disease is obese children.^16^ Childhood obesity is associated with hypertension, type 2 diabetes mellitus, dyslipidemia and premature death in adulthood.^17,18^ In the light of the increasing prevalence of childhood obesity worldwide, the effects of body mass index (BMI) on markers for ASCVD risk become increasingly important.^19^ It is incompletely understood whether Lp(a) serum levels in children are affected by factors such as BMI, age or sex. These questions are of clinical relevance because a stable biomarker in childhood would potentially allow for a general screening program in children and offer the possibility of reverse family screening to identify other family members at risk, such as parents, at an early age. We therefore examined Lp(a) serum concentrations in obese children of a large prospective population-based cohort and a healthy matched control group. In addition, we tested whether Lp(a) serum levels were associated with sex, age and cholesterol levels.

## Methods

### Study population and design

The prospective and longitudinal population-based Leipzig Research Center for Civilization Diseases (LIFE) childhood cohort study “LIFE Child” analyzes factors influencing the health and development of children in the city of Leipzig (Saxony, Germany).^20,21^

**Fig. 1:**
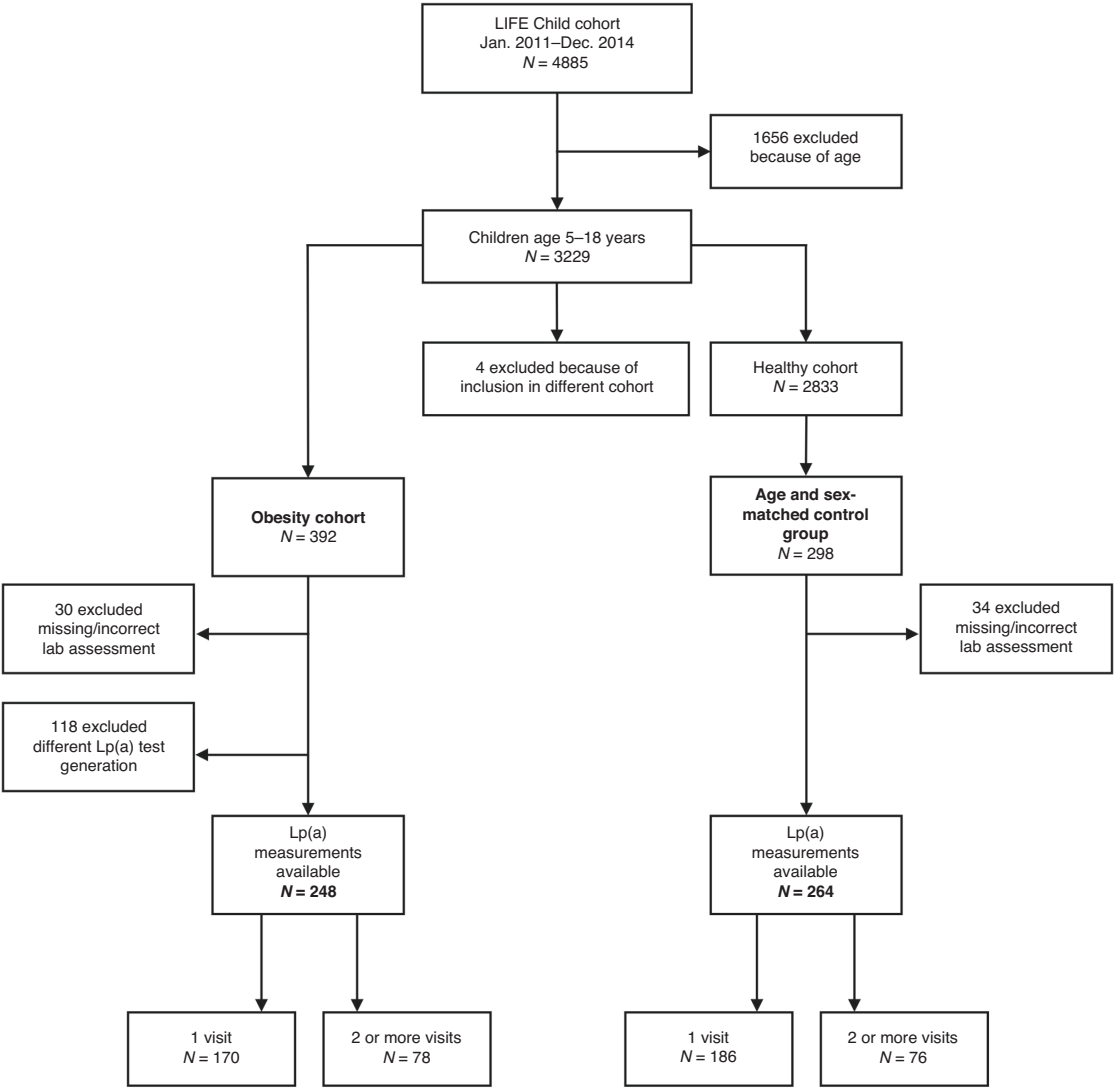
Selection of children with available Lp(a) value of the “LIFE Child” cohort. Flow chart of the recruitment for children enrolled in the Obesity cohort of the “LIFE Child” study and those who were part of the age- and sex-matched control group.

The children were recruited through advertising, media or word of mouth (for more detailed information visit www.life-child.de and refere to ref. ^22^). Healthy children were included in the study, while children with chronic, chromosomal and syndromic diseases were excluded. Children with a BMI >97th percentile of German age- and sex-specific norms were enrolled in the obesity cohort. Only children aged 5–18 years with at least one Lp(a) measurement using the Roche Cobas 6000/8000 platform (first generation) were included in the current analysis (Fig. 1). The data were collected between January 2011 and December 2014. Informed written consent was obtained from all parents and participants aged 12 years or older (Ethical approvement: Reg. No. 264-10-19042010). Study was registered at ClinicalTrials.gov under NCT02550236, and it adhered to principles of the Declaration of Helsinki. A total of 248 children from the “obesity cohort” were compared to 264 children from the healthy control cohort matched for age and sex. In total, *N* = 756 Lp(a) measurements from 512 children aged between 5 years and 18 years at enrollment were included in the analysis. At least 1 and up to 4 yearly follow-up visits (mean time between visits 12.6 months) were available for 154 children during which lipid profiles were assessed. No lipid profiles were available from children that refused blood collection or from whom no blood could be obtained.

### Sample pretreatment and analysis

Fresh venous blood samples (EDTA/ Serum monovettes: Sarstedt AG & Co, Germany) were drawn during the day following standardized protocols. Measurement of total cholesterol, low-density lipoprotein cholesterol (LDL-C), high-density lipoprotein cholesterol (HDL-C), triglycerides in serum was done in vitro by Roche/Hitachi on a Cobas 8000 Clinical Chemistry Analyzer by a validated specific homozygous enzymatic color test. Thus, LDL-C was directly measured not calculated. Triglyceride assay was not glycerol blanked. Apolipoprotein B (apoB) and apolipoprotein A1 (ApoA1) were determined by immunoassay (COBAS 6000/8000 lab analyzer system (Roche, Mannheim, Germany)). All measurements were carried out according to the manufacturer’s instructions.^23^ Whole mass Lp(a) plasma levels were measured using a particle-enhanced immunoturbidimetric assay on the Roche Cobas 6000/8000 platform (first generation).

Lp(a) risk categories were analyzed according to large meta- analyses and current guidelines for adults as cut-off to “rule out” (<30 mg/dL), interim gray zone (30 - <50 mg/dL) and cut-off to “rule-in” (≧ 50 mg/dL).^6,24,25^ Cut points for LDL-C were based on pediatric guidelines: LDL-C < 110 mg/dL (<3 mmol/l) is classified as acceptable, LDL-C values of 110–129 mg/dL (3–3.3 mmol/L) are classified as borderline high and LDL-C values > 130 mg/dL (>3.3 mmol/L) are defined as high. LDL-C values > 160 mg/dL (>4.1 mmol/L) were defined as suspectable of heterozygous familial hypercholesterolemia.^26^

### Anthropometric indicators

Body weight was assessed while children wore light underclothes to an accuracy of 50 g using a “Seca 701” calibrated electronic scale (Seca GmbH and Co. KG, Hamburg, Germany). Body height was measured to an accuracy of 0.1 cm using a “Dr. Keller I” stadiometer (Laengenmesstechnik GmbH Limbach-Oberfrohna, Germany). Body mass index was determined (weight in kilograms divided by measured height in meters squared) and converted to standard deviation scores (SDS) according to the German age- and sex-specific norms.^27^ Four weight groups were defined. A BMI-SDS below the 10th percentile (<1.282) was classified as underweight. Normal weight was defined as BMI-SDS ≥ −1.282 and ≤1.282 (≥10th percentile, ≤90th percentile). Those children with a BMI SDS > 1.282 and ≤1.881 (>90th percentile, ≤97th percentile) were classified as “overweight”; and those with a BMI- SDS > 1.881 (>97th percentile) as “obese”.^28,29^

### Statistical analyses

Descriptive statistics are presented as mean and standard deviation or median and IQR stratified by ASCVD risk groups based on Lp(a) levels (<30 and 30- <50 mg/dL and ≧50 mg/dL) to identify children with elevated or highly elevated Lp(a) levels. 22.9% of all Lp(a) values were below the lower limit of detection (LLD). For descriptive data presentation, they were set to the LLD value. For the analyses of the associations between Lp(a) as dependent variable and potential associated factors as well as between baseline Lp(a) and follow-up values, censored regression models^30^ for location, shape, and scale were applied^31^. Lp(a) values below LLD were marked as left censored. To account for the considerable skewness in Lp(a) values, Lp(a) values were log-transformed. Further, a Box-Cox-Power-Exponential distribution was assumed allowing for varying variance, skewness and kurtosis with varying covariates. We accounted for multiple measurements by setting corresponding weights. Associations were checked for non-linearity and interactions with age and sex. *P*-values of ≤ 0.05 were considered significant. Stata (Version 16; StataCorp LP) and GraphPad Prism Version 9.4.0 and R (Version 4.3.1)^32^ were used for statistical analysis.

## Results

**Fig. 2:**
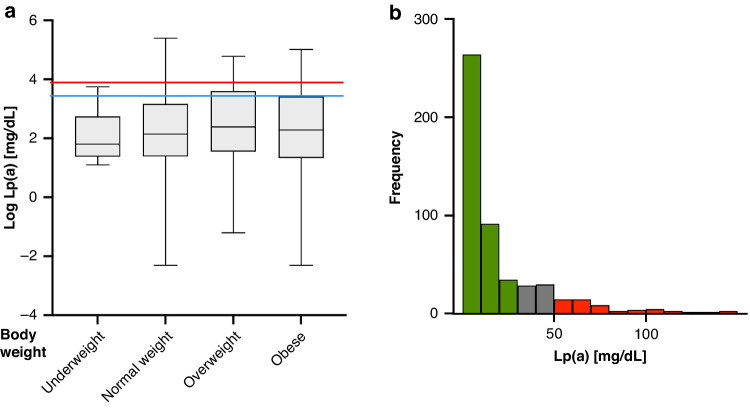
Distribution of Lp(a) values in children stratified by body mass index. **a** Log Lp(a) serum concentrations over body weight (BMI SDS), *n* = 512. Blue line indicates Lp(a) 30 mg/dL, red line indicates Lp(a) 50 mg/dL. Boxes indicate SEM, bars indicate min. to max. values. *P < 0.05. **b** Distribution of Lp(a) serum concentrations in children (n = 512, age 5–18 years. Green: Lp(a) < 30 mg/dL, gray: Lp(a) ≧ 30 mg/dL - <50 mg/dL, red: Lp(a) ≧ 50 mg/dL).

The clinical characteristics of the children are depicted in Table 1. Children with obesity had slightly higher LDL-C levels. Lp(a) levels did not differ between the obese and the non-obese cohort. Moreover, Lp(a) did not correlate with BMI-SDS (*n* = 498, ß = 0.004, *P* = 0.49) (Fig. 2a). Since BMI showed no correlation with Lp(a) serum concentrations, the subsequent analyses were performed for the total cohort (n = 512). The distribution of Lp(a) concentrations were right-skewed, similar to the distribution in adults (Fig. 2b).

**Table 1:** Clinical characteristics at enrollment of participants of the obesity cohort and of the matched healthy cohort.

At enrollment	Obesity cohort	Healthy cohort	*P*
*n*	248	264	
Female, %	46.7	50.7	0.37
Age, yrs	11.5 (2.8, 248)	11.7 (2.8, 264)	0.52
BMI, SDS	2.4 (0.5, 239)	0.02 (1.0, 259)	<0.0001
Laboratory parameters			
Lp(a), mg/dL	9.4 (3.9–32.1, 248)	9.8 (4.0–28.4, 264)	0.51
Total cholesterol, mmol/L	4.2 (0.7, 128)	4.1 (0.7, 264)	0.02
LDL-C, mmol/L	2.5 (0.6, 181)	2.3 (0.6, 263)	<0.0001
HDL-C, mmol/L	1.3 (0.3, 183)	1.6 (0.3, 263)	<0.0001
Triglycerides, mmol/L	1.1 (0.6, 183)	0.8 (0.4, 263)	<0.0001
apoB, g/L	0.8 (0.2, 248)	0.7 (0.2, 263)	<0.0001
ApoA1, g/L	1.3 (0.2, 248)	1.4 (0.2, 263)	<0.0001

Data is presented as: Mean (standard deviation, number of available records) or median with interquartile range (1st quartile–3rd quartile) is presented for Lp(a).

*Yrs* years, *BMI* body mass index, *SDS* standard deviation scores, *Lp(a)* lipoprotein(a), *LDL-C* low density lipoprotein cholesterol, *HDL-C* high density lipoprotein cholesterol, *apoB* apolipoprotein B, *ApoA1* apolipoprotein A1.

### Distribution of Lp(a) serum concentrations in children

The clinical characteristics of all children at enrollment stratified by Lp(a) risk status are presented in Table 2. The median Lp(a) level at enrollment was 9.7 mg/dL (IQR 4.0–28.3). A total of 76.0% of children had Lp(a) levels <30 mg/dL. Lp(a) concentrations between 30- <50 mg/dL were detected in 11.5% while 12.5% of children had Lp(a) levels ≧50 mg/dL.

**Table 2:** Clinical characteristics of children at enrollment for all and stratified by lipoprotein(a) levels < 30 mg/dL, 30- < 50 mg/dL and ≧50 mg/dL.

At enrollment	All	Lp(a) < 30 mg/dL	Lp(a) 30 mg/dL- < 50 mg/dL	Lp(a) ≧ 50 mg/dL
*n* (%)	512 (100)	389 (76.0)	59 (11.5)	64 (12.5)
Female, %	51.2	49.6	35.6	56.3
Age, yrs	11.6 (2.8; 512)	11.5(2.8, 389)	11.5 (2.7, 59)	12.7 (2.7, 64)
BMI, SDS	1.2 (1.4, 498)	1.1 (1.4, 381)	1.2 (1.4, 54)	1.5 (1.2, 63)
Laboratory parameters				
Lp(a), mg/dL	9.7 (4.0–28.3, 512)	6.3 (3.1–11.6, 389)	39.4 (34.2–44.1, 59)	70.0 (59.6–100.5, 64)
Total cholesterol, mmol/L	4.1 (0.7, 446)	4.1 (0.7, 339)	4.1 (0.7, 52)	4.4 (0.7, 55)
LDL-C, mmol/L	2.4 (0.6, 444)	2.4 (0.6, 337)	2.4 (0.6, 52)	2.7 (0.6, 55)
HDL-C, mmol/L	1.5 (0.4, 446)	1.5 (0.4, 339)	1.4 (0.3, 52)	1.4 (0.3, 55)
Triglycerides, mmol/L	0.9 (0.5, 446)	0.9 (0.5, 339)	0.9 (0.5, 52)	1.0 (0.5, 55)
apoB, g/L	0.7 (0.2, 511)	0.7 (0.2, 388)	0.7 (0.2, 59)	0.8 (0.2, 64)
ApoA1, g/L	1.4 (0.2, 511)	1.4 (0.2, 388)	1.4 (0.2, 59)	1.3 (0.2, 64)

Data is presented as: Mean (standard deviation, number of available records), median with interquartile range (1st quartile–3rd quartile) is presented for Lp(a).

*Yrs* years, *BMI* body mass index, *SDS* standard deviation scores, *Lp(a)* lipoprotein(a), *LDL-C* low density lipoprotein cholesterol, *HDL-C* high density lipoprotein cholesterol, *apoB* apolipoprotein B, *ApoA1* apolipoprotein A1.

### Associations of Lp(a) levels with age, sex, and lipid levels

**Fig. 3:**
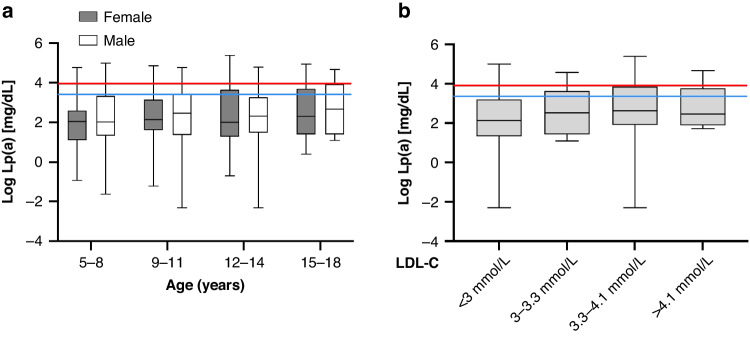
Association of Lp(a) values with age, sex and LDL-cholesterol. **a** Distribution of log Lp(a) serum concentrations over age in female (gray) and male (white) children and (**b**) over LDL-cholesterol at enrollment. Blue line indicates Lp(a) 30 mg/dL, red line indicates Lp(a) 50 mg/dL. Boxes indicate SEM, bars indicate min. to max. values. *P < 0.05.

Lp(a) levels did not differ between male and female children (*n* = 512, ß = −0.01, *P* = 0.51) (Fig. 3a). Therefore, sexes were combined for the subsequent analyses. There was no significant association between Lp(a) concentrations and age (*n* = 512, ß = 0.004, *P* = 0.12) (Fig. 3a). Lp(a) levels were associated positively with LDL-C levels (*n* = 444, ß = 0.05, *P* < 0.0001) (Fig. 3b), total cholesterol (*n* = 446, ß = 0.04, *P* = 0.002) and apoB (*n* = 511, ß = 0.12, = 0.006). Since Lp(a) is an apoB-containing protein^6^, this observation likely reflects the contribution of Lp(a) to the respective pools. For TC and LDL-C, these correlations were observed in girls and in boys while the associations were stronger in girls (total cholesterol: male: ß = 0.03, *P* = 0.02; female: ß = 0.06; *P* < 0.001. LDL-C: male ß = 0.03; *P* = 0.02; female: ß = 0.07; *P* < 0.001). For apoB, the association was rather strong in females (ß = 0.20, *P* < 0.001) but did not reach statistical significance in male (ß = 0.05, *P* = 0.386). HDL-C (*n* = 446, ß = −0.01, *P* = 0.28), triglycerides (*n* = 446, ß = 0.1, *P* = 0.24), and ApoA1 (*n* = 511, ß = −0.13, *P* = 0.46) were not significantly associated with Lp(a), neither across the whole sample nor stratified by sex or after adjusting for BMI-SDS.

### Intra-individual variability of Lp(a) values in repeat measurements over time

In 154 children repeat measurements of Lp(a) were available (1–4 follow up measurements, mean time between annual follow up visits: 12.6 months (standard deviation (SD) 0.2), range of 9.5 months to maximal 18 months between follow up visits). In total, 2 measurements of Lp(a) per individual were available in 154 children, 3 measurements of Lp(a) per individual were available in 52 children and 4 repeat measurements in 6 children. Mean Lp(a) values did not differ between the first and second visit (ß = 0.02, *P* = 0.623). This was also true for the second and third visit (ß = 0.02, *P* = 0.600). The association between repeated Lp(a) values per individual was independent of BMI. Furthermore, a correction for BMI did not alter the coefficient significantly.

We analyzed whether repeat Lp(a) measurements placed individuals in different Lp(a) risk categories.^27^ This was true for 6.5% (*n* = 10) of all children with repeat measurements. A percentage of 1.9% of all children (*n* = 3) showed one value in the interim gray zone (≧30 mg/dL <50 mg/dL) and one value in the “rule-in” category (≧50 mg/dL) in repeat measurements with a mean standard deviation between repeated values of 7.9 ± 5.4 mg/dL. In 3.3% of children (*n* = 6), Lp(a) values changed from the “rule-in” category (<30 mg/dL) to the interim gray zone (≧30 mg/dL <50 mg/dL) with a mean standard deviation between repeated values of 5.6 ± 3.5 mg/dL, indicating that the values were clustering around the cut-off value of 30 mg/dL. In 1 child, one measurement <30 mg/dL and one measurement ≧50 mg/dL was observed.

## Discussion

The principal findings of this population study indicate that lipoprotein(a) concentrations were independent of BMI, age and sex during childhood. Obesity did not have a significant effect on Lp(a) levels. These findings pave the way for further studies to evaluate the potential use of Lp(a) in children and young adults for risk assessment and reverse family screening.

Observational and genetic evidence have identified lipoprotein(a) as causal factor for ASCVD and aortic stenosis in adults.^3,13^ Lp(a) values predict long-term cardiovascular outcome and improve risk prediction, making measuring Lp(a) valuable in ASCVD risk assessment in adults.^33^ Lp(a) levels are relatively stable in adulthood.^13^ The ESC/EAS guidelines recommend measuring Lp(a) once in a lifetime in adults.^7^ However, data on the distribution of lipoprotein(a) in children is limited.^8–10,34^ Our study shows that Lp(a) values are independent of sex, age and BMI. These findings add to the findings of previous studies.^8,35^ Gannagé-Yared et al. report an association of Lp(a) with BMI in Lebanese school children.^8^ This finding was not confirmed in our data. No association was observed between Lp(a) a body mass index in either the obesity cohort nor in the healthy cohort. Additionally, when combining the two cohorts Lp(a) was not affected by high BMI levels. It’s important to note that Lp(a) concentrations are influenced by ethnicity, and in our study, most of the children were Caucasian and had higher body weight compared to the Lebanese population, making a direct comparison between the two populations challenging.^6^

De Boer et al., report an 1.5-fold increase of Lp(a) values from age 9 to 14.^9^ However, it is important to note that their study involved a analysis of a selected group of children treated in lipid clinics, with the majority having genetically proven heterozygous familial hypercholesterolemia (FH) and a significant portion receiving lipid-lowering medication. Therefore, these children substantially differed from our population-based cohort of healthy children.^9^ The majority of children in our analysis had very low Lp(a) values. Lp(a) quantification assays are rarely designed to quantify very low Lp(a) values. Due to analytical issues, Lp(a) values can differ slightly between repeated measurements. Thus, we focused on changes in the categories for Lp(a)-associated cardiovascular risk. It is important to note that the cut-off values for Lp(a) have not been analyzed in children yet and are an extrapolation from adult literature. We demonstrate that adjudication to Lp(a) risk categories remains constant per individual with increasing age, suggesting that the Lp(a) risk category in childhood likely reflects the risk category in adulthood.

In the absence of specific Lp(a) lowering drugs, the purpose of Lp(a) measurements is the risk assessment and the family screening. In the 2022 EAS Lp(a) consensus paper, a pragmatic “rule-in” or “rule out” approach is recommended.^6^ Therefore, the clinically important information is whether the Lp(a) concentrations change between these two categories and how Lp(a) levels develop in the interim gray zone. Our analysis supports the concept that one measurement is sufficient to identify the children that are in the ‘rule-in’ category. While the cardiovascular risk associated with Lp(a) is apparent^6^, clinical evidence of better cardiovascular outcome of adults with detection of high Lp(a) during childhood is still pending.

In two large, longitudinal, prospectives studies, the YFS (Cardiovascular Risk in Young Finns Study) and the BHS (Bogalusa Heart Study) study, participants who were exposed to Lp(a) levels ≥30 mg/dL in youth had a at least a 2.0 times greater risk of developing ASCVD in adulthood in comparison to children with Lp(a) levels <30 mg/dl.^36^ These findings emphasize that the goal must be to reduce the cumulative effect of LDL-C and other apoB-containing lipoproteins on the risk of ASCVD as early as possible, as it seems that a significant part of this burden is acquired during childhood and adolescents.^37^ Interestingly, no association between high Lp(a) levels and increased carotid intima-media thickness (cIMT) was observed in the this study despite the increased risk for ASCVD.^36^ In line with this data, in healthy children and hypercholesterolemic children, no association between arterial wall thickening and levels of Lp(a) was detected.^38,39^ This raises the question of whether cIMT is a suitable marker for Lp(a) mediated vascular damage.^36,38^ Children of the obese cohort were characterized by higher lipid values even though no sigificant correlation was observed between BMI and Lp(a). With regard to the overall high cardiovascular risk associated with childhood obesity early detection of additional cardiovascular risk factors such as Lp(a) seems even more important. Knowing high Lp(a) value from an early age can help motivate healthy behavior and better control of other cardiovascular risk factors.^40^ Primordial and primary prevention are key to allow children to reach adulthood with the lowest cardiovascular burden possible.^37,41^ This is especially important in obese children.

In the future, the treatment of Lp(a) with specific and potent Lp(a) lowering agents that are currently in clinical development will likely require repeated measurements. In the meantime, the safety and efficacy of Lp(a)-lowering drugs in children need to be established and joint efforts are needed to standardize Lp(a) testing.^3,42^

Accumulating evidence supports elevated Lp(a) levels in youth as an indepent risk factor for premature ASCVD.^36,43^ Therefore, the inclusion of Lp(a) screening as part of cardiovascular risk assessment in children should be reconcidered.^44^ Furthermore, a strong argument for a potential universal screening of children for Lp(a) is the reverse or cascade screening.^45^ Identification of children with high Lp(a) offers the important opportunity to provide cardiovascular prevention to the affected parent and additional relatives. At present, the general screening rate for cholesterol in children is very low and testing for Lp(a) in children is even lower.^45–47^ Therefore, this study may be helpful to demonstrate that Lp(a) measurement in children meets the criteria for a successful screening strategy as proposed by e.g., Wilson and Jungner^48,49^.

A population that may especially benefit from Lp(a) testing are individuals with familial hypercholesterolemia. In patients with heterozygous famial hypercholesterolemia (HeFH) the frequency of elevated Lp(a) levels is very high, ranging from 30 to 50%.^50^ The combined cumulative lifelong exposure to two genetically determined pro-atherogenic lipoproteins, LDL-C and Lp(a), is a potent driver of ASCVD in HeFH patients. In children with familial hypercholesterolaemia, Lp(a) concentrations have been identified as an independent and additional risk factor for arterial wall thickening during a follow up period until adulthood.^43^ In HeFH children, Lp(a) values may be more predictive of premature ASCVD events than LDL-C levels.^51^ Therefore, our data support the concept of combining the universal screening for HeFH and for Lp(a) in children.^9,43,45,52^ Furthermore, in children with probable FH but no detected genetic mutation, elevated Lp(a) values may be the underling cause for the clinical presentation of FH, as shown by de Boer et al., and might lead to improved accuracy of FH diagnosis.^53,54^ Still, potential harm, such as psychological or financial implications, which might arise from identifying a large proportion of children at a young age with elevated Lp(a) values, need to be taken into consideration as long as clinical evidence on outcomes is pending.^40^

### Strengths and limitations

Strengths of this study include the number of children in the analyzed cohort, which is representative of the German population, and the availability of multiple measurements of Lp(a) per individual in a similar time interval performed in one central laboratory. However, all the important limitations of a retrospective analysis apply. Lp(a) serum concentrations were measured in the obesity subgroup and a normal weight control group matched for age and sex of the “LIFE Child” cohort. The time span between repeated measurements was relatively short with a mean of 12.6 months (SD 0.2), range of 9.5 months to maximal 18 months between follow up visits. The dataset is limited in size of repeated measurements per individual. The population was primarily Caucasian, and serum concentrations may differ in children with other ethnic backgrounds. The cholesterol content of Lp(a) particles is included in LDL-C concentrations reported by LDL-C assays. Lp(a) particles vary in apo(a) isoforms. An adjustment of the correction factor for the isoform size is currently not possible.^55^ Furthermore, a high inter- and intraindividual variation of Lp(a) cholesterol (Lp(a)-C) relative to Lp(a) mass was observed by Yeang et al.^56^. According to the current EAS consensus paper of Kronenberg et al., routine correction of LDL-C for Lp(a)-C is not recommended.^6^ The observed association of total cholesterol, apoB and LDL-C with Lp(a) values in our study is likely due to the contribution of Lp(a)-C to the respective pools. Additionally, a limitation of our study is the lack of association of Lp(a) values with vascular measures, such as cIMT.

## Conclusion

The data of the present study show that Lp(a) values are independent of BMI and sex. Lp(a) risk categories remain stable over time and throughout childhood. Therefore, measurement of Lp(a) in childhood may be useful for identifying both children and family members at high and very high cardiovascular risk. Future studies are needed to assess whether systematic measurement of LDL-C and Lp(a) in children represents a strategy to reduce the life-long burden of ASCVD by implementing cardiovascular prevention before manifestation of vascular pathologies.

## Data Availability

The datasets generated during and/or analysed during the current study are available from the corresponding author on reasonable request.
